# Combretastatin-A4 phosphate improves the distribution and antitumor efficacy of albumin-bound paclitaxel in W256 breast carcinoma model

**DOI:** 10.18632/oncotarget.11249

**Published:** 2016-08-12

**Authors:** Meng Gao, Dongjian Zhang, Qiaomei Jin, Cuihua Jiang, Cong Wang, Jindian Li, Fei Peng, Dejian Huang, Jian Zhang, Shaoli Song

**Affiliations:** ^1^ Laboratory of Translational Medicine, Jiangsu Province Hospital of Integrated Traditional Chinese and Western Medicine, Nanjing University of Chinese Medicine, Nanjing 210028, Jiangsu Province, P.R.China; ^2^ Laboratory of Translational Medicine, Jiangsu Province Academy of Traditional Chinese Medicine, Nanjing 210028, Jiangsu Province, P.R.China; ^3^ Department of Natural Medicinal Chemistry and State Key Laboratory of Natural Medicines, China Pharmaceutical University, Nanjing 210009, Jiangsu Province, P.R. China; ^4^ Department of Nuclear Medicine, Renji Hospital, Shanghai Jiaotong University, School of Medicine, Shanghai 200127, P.R. China

**Keywords:** combretastatin-A-4 phosphate, interstitial fluid pressure, albumin-bound paclitaxel, tissue distribution, combination therapy

## Abstract

Nanomedicine holds great promise for fighting against malignant tumors. However, tumor elevated interstitial fluid pressure (IFP) seriously hinders convective transvascular and interstitial transport of nanomedicines and thus damages its antitumor efficacy. In this study, combretastatin-A4 phosphate (CA4P) was utilized to reduce tumor IFP, and thereby to improve the intratumoral distribution and antitumor efficacy of nanoparticle albumin-bound paclitaxel (nab-paclitaxel). IFP was measured using the wick-in-needle method in tumors growing subcutaneously pretreatment and posttreatment with a single intravenous injection of CA4P. The tracing method of iodine 131 isotope was used for biodistribution analysis of nab-paclitaxel. Liquid chromatography coupled with tandem mass spectrometry was used to detect the intratumoral concentration of paclitaxel. Magnetic resonance imaging was applied to monitor tumor volume and ratios of necrosis. The tumor IFP continued to decline gradually over time following CA4P treatment, reaching approximately 31% of the pretreatment value by 1 h posttreatment. Biodistribution data indicated that both ^131^I-nab-paclitaxel and paclitaxel exhibited higher tumor uptake in CA4P + ^131^I-nab-paclitaxel group compared with I^131^-nab-paclitaxel group. Nab-paclitaxel combined with CA4Pshowed significant tumor growth inhibition and higher tumor necrosis ratio relative to PBS, CA4P and nab-paclitaxel group, respectively. In conclusion, CA4P improved the intratumoral distribution and antitumor efficacy of nab-paclitaxel in W256 tumor-bearing rats.

## INTRODUCTION

A major challenge in chemotherapy is to change the biodistribution of drugs to reduce free drug toxicity and favor tumor accumulation. Nanomedicines have been designed to overcome this challenge, based on the following advantages: (a) improve the solubility of hydrophobic compounds, (b) protect a molecule from undesirable interactions with biological milieu components and improve its stability, (c) provide controlled release of the contents, and (d) favorably alter the pharmacokinetics and biodistribution [1–3]. One of the nanomedicines that are used clinically in the treatment of cancer is nanoparticle albumin-bound paclitaxel (brand name: Abraxane), which showed less toxic and superior efficacy compared with polyethylated castor oil-based paclitaxel in the treatment of breast cancer [4]. Although high expectations have been placed on that nanomedicines would change the current status quo of cancer treatment, the increase in overall survival of cancer patients is only modest in many cases [5].

Transport of a therapeutic agent from the systemic circulation to cancer cells is a three-step process. First, nanoparticles flow to different regions of tumors via blood vessels. They must then cross the vessel wall, and finally, penetrate through the interstitial space to reach the target cells [5]. The concerted effects of the lack of functional lymphatic vessels and the vascular hyperpermeability result in the elevated interstitial fluid pressure (IFP) [6]. Although nanomedicines could be preferentially delivered to tumors owing to the enhanced permeability and retention effect, the convective transvascular and interstitial transport for them is severely hampered due to the elevated IFP [3, 7], which leads to a poor penetration into and distribution across the interstitium [7]. Therefore, lowering IFP in solid tumors may be a great strategy to improve the uptake and distribution of nanomedicines in tumors.

Many efforts have been made to demonstrate the feasibility of this strategy. For instance, collagenase-induced reductions in IFP can restore convective mass transport, thereby improving the uptake and distribution of monoclonal antibodies in human osteosarcoma xenografts [8]. Similarly, hyaluronidase-induced reductions in IFP can enhance transvascular transport of nanoprobes, thereby improving the uptake and distribution of liposomal doxorubicin in the same model [9]. Other agents that have been used to lower tumor IFP include bevacizumab, tumor necrosis factor-alpha, dexamethasone, prostaglandin E1-methyl ester, labradimil, docetaxel, nicotinamide, etc. [3, 10] However, all of these agents have some limitations, including toxicity to normal tissue or lack of tumor selectivity.

Recently, vascular-disrupting strategies have been explored to lower tumor IFP. Vascular disrupting agents (VDAs) are a relatively new class of drugs that target the established tumor vasculature and cause a rapid and selective vascular shutdown and subsequent necrosis in the tumor core [11]. VDAs preferentially target the intratumoral vascular bed due to its fragile phenotype compared to normal tissue blood vessels [12]. One of the most promising VDAs is combretastatin-A4 phosphate (CA4P), which can induce 90–99% of tumor necrosis at well tolerated doses and is currently in phase 3 clinical trials [13]. It has been demonstrated that CA4P could significantly decrease IFP in both C3H mammary carcinomas and KHT sarcomas [14, 15].

Based on the above, we postulated that CA4P might improve the uptake and distribution of nab-paclitaxel in tumor by reducing IFP, thereby enhancing the antitumor efficacy of nab-paclitaxel. Meanwhile, the combination of CA4P and nab-paclitaxel may provide a synergistic and complementary antitumor effect, in which CA4P could induce the destruction of large areas of the interior of tumors while nab-paclitaxel could effectively kill residual tumor cells in the outer layer.

To test this hypothesis, IFP was measured by the wick-in-needle technique after i.v. injection of CA4P in rats bearing W256 breast carcinoma. The whole-body biodistribution and tumor distribution of ^131^I-nab-paclitaxel injected i.v. at 1 h after CA4P injection was studied by gamma counting and autoradiography, respectively. The intratumoral uptake of paclitaxel (PTX) was determined using liquid chromatography coupled with tandem mass spectrometry (LC-MS/MS). Finally, the anticancer efficacy of nab-paclitaxel combined with CA4P was evaluated in the same model by magnetic resonance imaging (MRI).

## RESULTS

### Radiolabeling and *in vitro* stability

In order to facilitate the biodistribution study, nab-paclitaxel was radiolabeled with iodine 131. The thin layer chromatography (TLC) determination showed that the radiochemical purity (RCP) of ^131^I-nab-paclitaxel was greater than 95% after purification, and *in vitro* stabilities of ^131^I-nab-paclitaxel incubated in rat serum at 37°C were excellent with RCP over 92% up to 24 h.

### CA4P reduced tumor IFP

**Figure 1:**
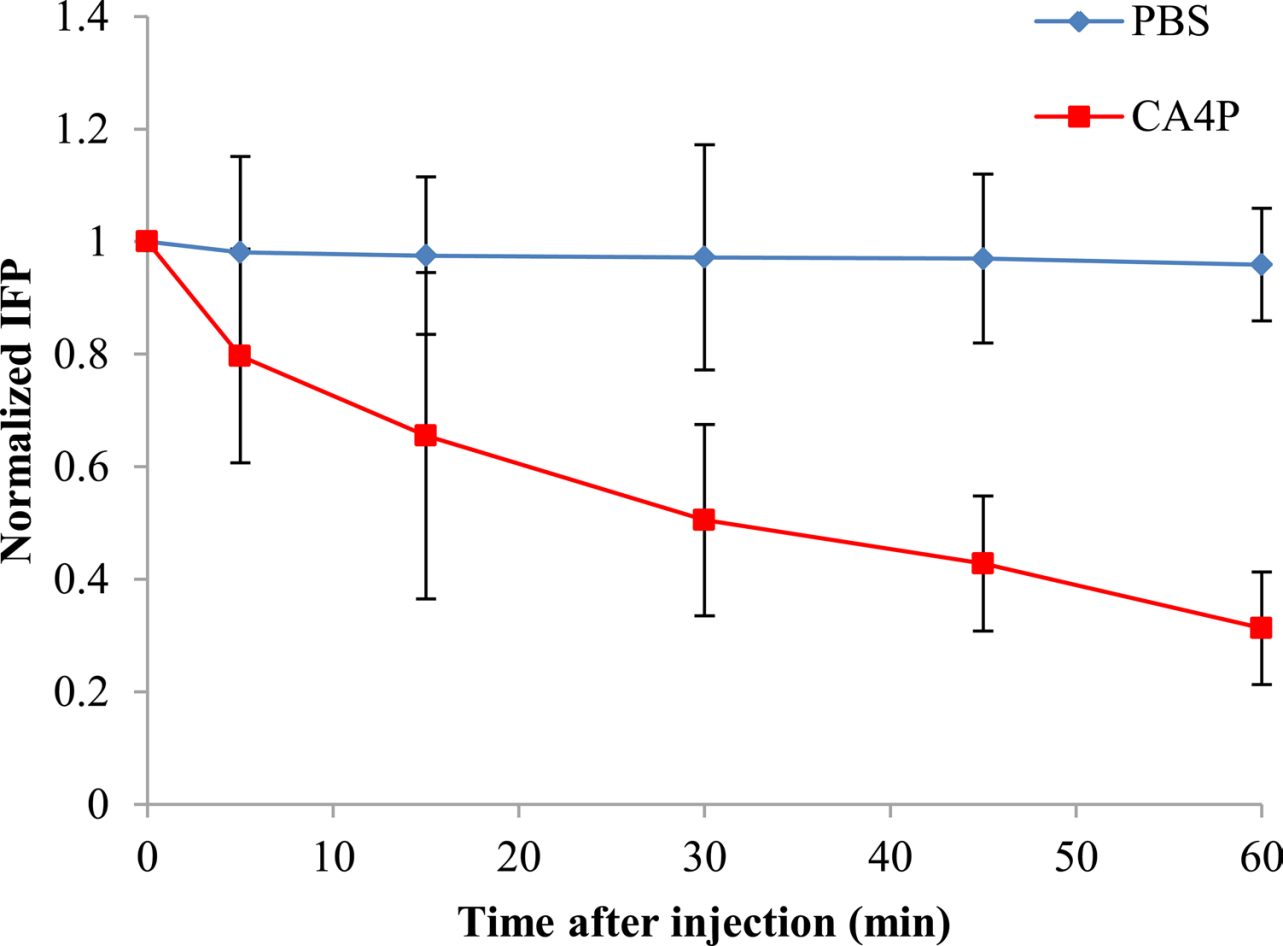
IFP response as a function of time after i.v injection of PBS or CA4P (30 mg/kg). IFP values were normalized to the pressure values before PBS or CA4P injection. Each value is the mean of n tumors, the error bars represent SD.

Figure 1 shows the temporal changes in IFP following administration of CA4P with the values normalized to the pretreatment value for each tumor. Tumors treated with 30 mg/kg of CA4P showed IFP levels continued to decline gradually over time, reaching approximately 31% of the initial pretreatment value by 60 min post injection. Tumors treated with PBS did not induce any significant reduction in IFP.

### Whole-body biodistribution and intratumoral distribution of ^131^I-nab-paclitaxel

**Figure 2:**
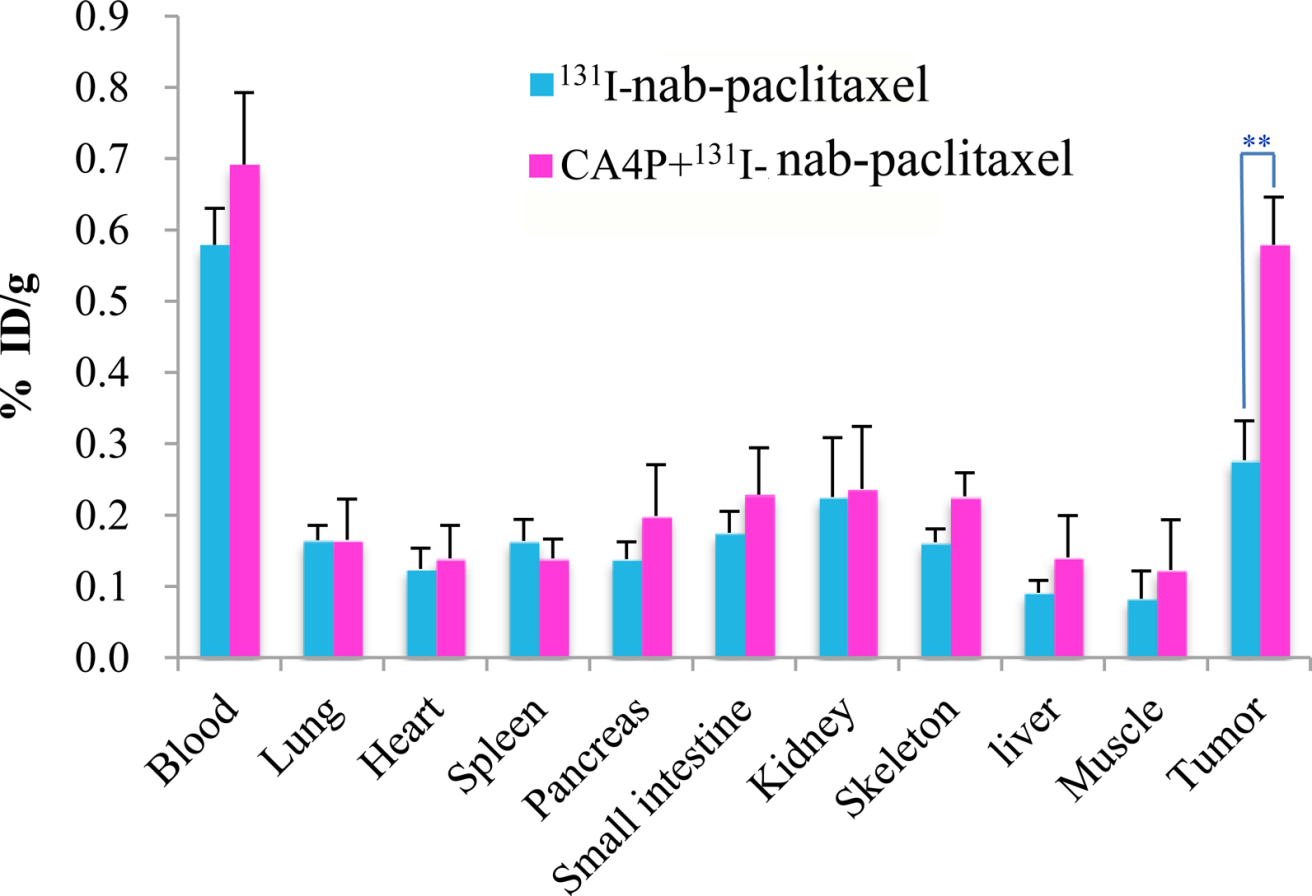
Biodistribution of ^131^I-nab-paclitaxel inW256 tumor-bearing rats at 24 h post injection in ^131^I-nab-paclitaxel and CA4P + ^131^I-nab-paclitaxel group Data are expressed as percentage injected dose per gram of tissue (%ID/g). **P < 0.01.

The whole-body biodistribution of ^131^I-nab-paclitaxel at 24 h post injection is shown in Figure 2. ^131^I-nab-paclitaxel exhibited significantly higher tumor uptake in CA4P + ^131^I-nab-paclitaxel group compared with^131^I-nab-paclitaxel group (0.58 ± 0.07% ID/g vs 0.28 ± 0.06% ID/g, *p* < 0.01). In all normal organs or tissues, the uptake of ^131^I-nab-paclitaxel had no significant difference between the ^131^I-nab-paclitaxel group and CA4P + ^131^I-nab-paclitaxel group (*p* > 0.05). This shows that CA4P can significantly improve tumor uptake of ^131^I-nab-paclitaxel without affecting the biodistribution of ^131^I-nab-paclitaxel in normal organs or tissues.

**Figure 3:**
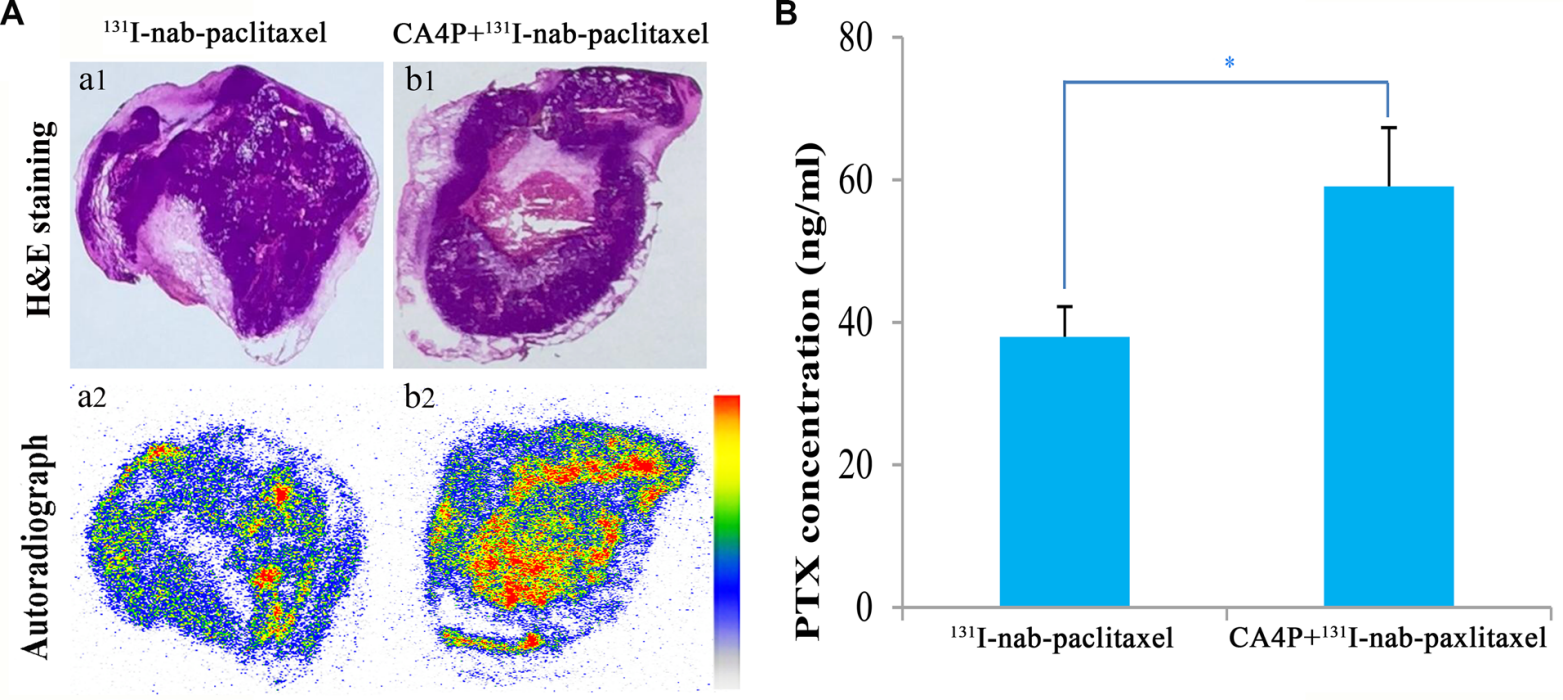
Representative autoradiographs (a2, b2) and corresponding H&E images (a1, b1) of 30 μm tumor slices from W256 tumor-bearing rats (A) and the intratumoral distribution of paclitaxel (B) at 24 h post i.v of ^131^I-nab-paclitaxel (14.8 MBq/kg of ^131^I-nab-paclitaxel, 6 mg/kg of nab-paclitaxel) in ^131^I-nab-paclitaxel and CA4P + ^131^I-nab-paclitaxel group. Data represents the mean ± SD. *P < 0.05.

Representative autoradiographs of tumor slices from ^131^I-nab-paclitaxel group and CA4P + ^131^I-nab-paclitaxel group are displayed in Figure 3A. Tumor uptake was significantly higher in CA4P + ^131^I-nab-paclitaxel group than that in ^131^I-nab-paclitaxel group, which is consistent with the results of the gamma counting. The intratumoral distribution of ^131^I-nab-paclitaxel in ^131^I-nab-paclitaxel group was primarily localized in the periphery of the tumor, while in CA4P + ^131^I-nab-paclitaxel group, ^131^I-nab-paclitaxel penetrated further into the tumor center and its intratumoral distribution is relatively more uniform. This shows that CA4P can not only improve tumor uptake of ^131^I-nab-paclitaxel, but also increase its penetration into tumor interior.

### Quantitation of intratumoral PTX

The quantitation of intratumoral PTX at 24 h post injection is presented in Figure 3B. CA4P + ^131^I-nab-paclitaxel group showed higher PTX concentration in the tumor compared with ^131^I-nab-paclitaxel group (59.08 ± 8.26 ng/ml vs 37.98 ± 4.23 ng/ml, *p* < 0.05), which indicated that CA4P improved tumor uptake of PTX.

### *In vivo* MRI

**Figure 4:**
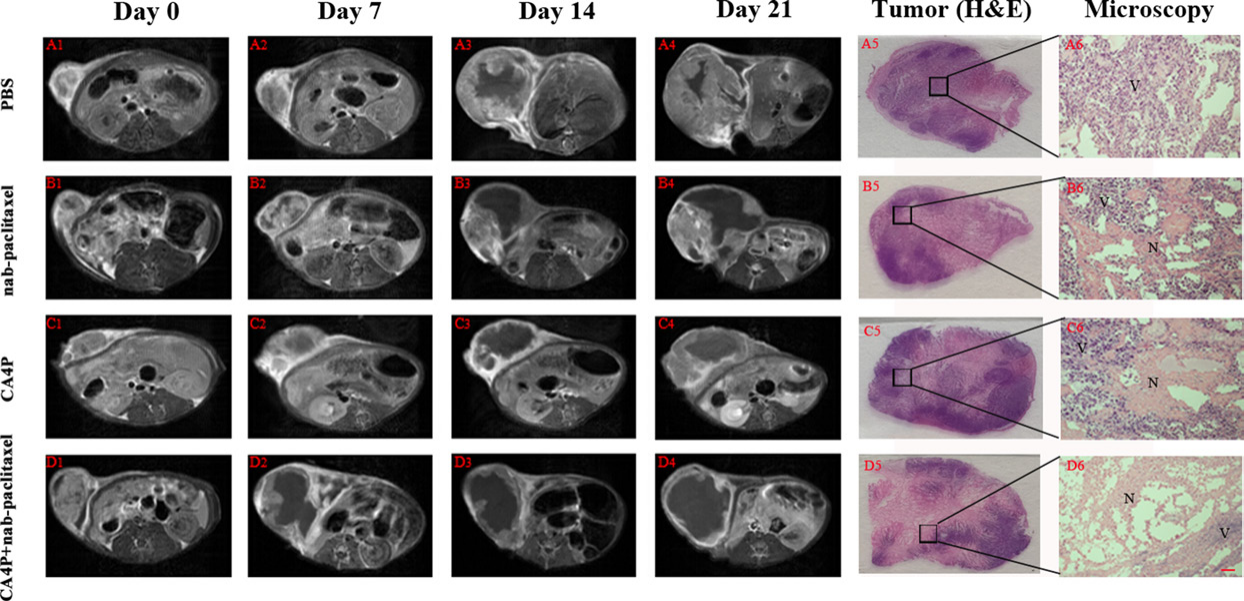
Contrast enhanced T1 (CE-T1) MR images of representative tumor bearing rats from 4 groups at day 0 (A1-D1), 7 (A2-D2), 14 (A3-D3), 21 (A4-D4) Tumors in PBS, nab-paclitaxel and CA4P group grew faster than that of CA4P + nab-paclitaxel group. Macroscopic photographs (A5–D5) showed extensive central necrosis surrounded by viable tumor tissues in the three therapy groups. Microscopic photographs (A6–D6) showed the interface between necrotic (N) and viable (V) tumor tissues in the three therapy groups. Scale bar = 50 μm.

Tumors at baseline appeared slightly hypointense or isointense on T1W images and hyperintense on T2W images. Contrast enhancement was observed on CE-T1Wimages, suggesting hypervascularity of the subcutaneous W256 mass. A nonenhanced central region surrounded by a thin rim enhancement presented on CE-T1W images in CA4P + nab-paclitaxel group after sequential i.v. administrations of CA4P and nab-paclitaxel, suggesting the presence of massive necrosis and minimum tumor residue (Figure 4D2–4D4), which differed from the tumors with spontaneous necrosis in PBS group (Figure 4A2–4A4). This was speculatively attributed to the synergistic effect of CA4P and nab-paclitaxel. The tumor growth in CA4P + nab-paclitaxel group was well inhibited and maintained a stable in size until the endpoint. By contrast, the tumors in the other three groups grew rapidly with the tumor size progressively becoming larger. Irregularly enhanced thick rims suggestive of viable tumor tissue were shown in nab-paclitaxel group and CA4P group at later time points. Necrotic and viable tumor could be determined by histopathological findings (Figure 4A5–4D5, 4A6–4D6). Necrosis occupied a large-area of the tumor mass at the endpoint in all groups except for the PBS group.

### Tumor volume and necrosis ratio

**Figure 5:**
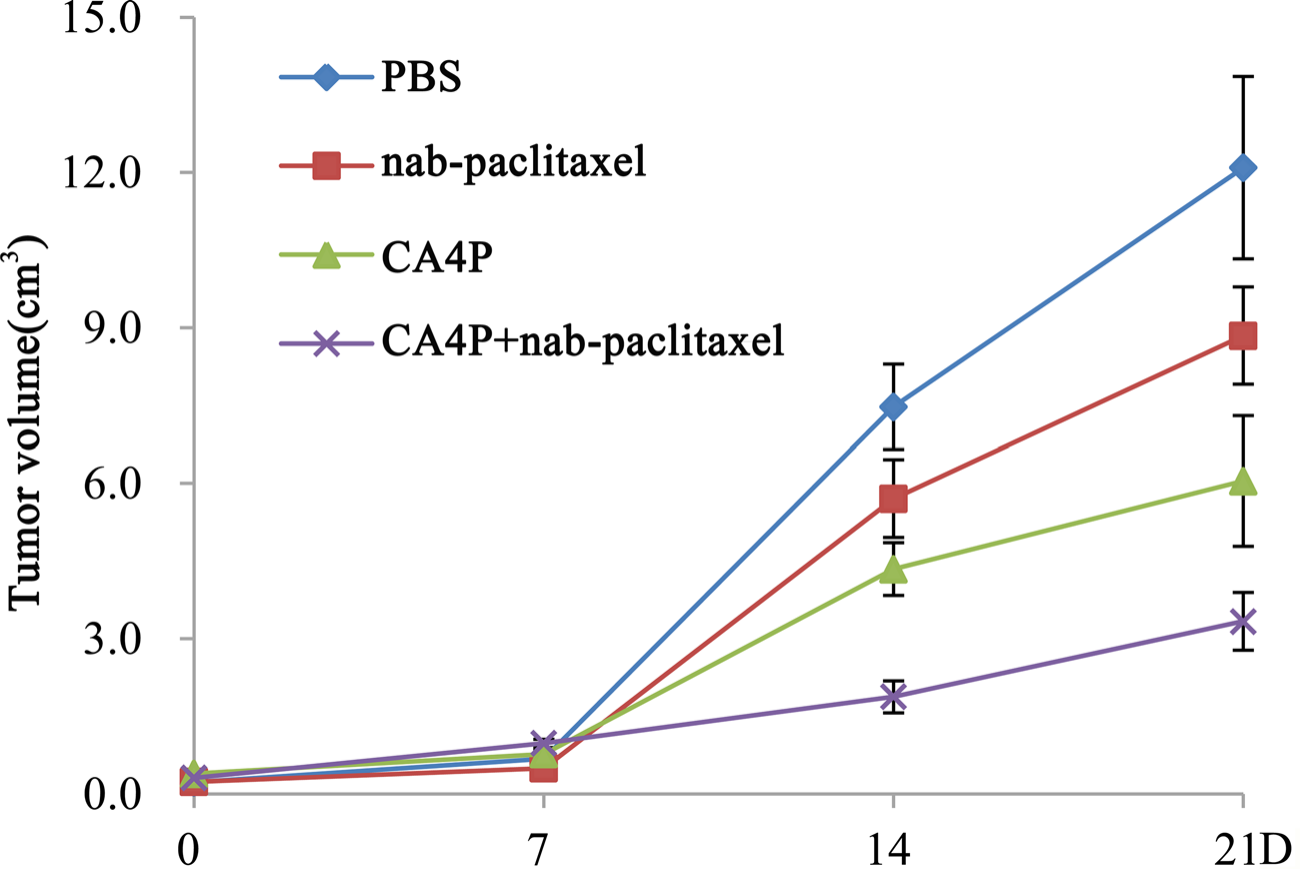
Tumor growth curve on day 0, 7, 14 and 21 post-therapy Significant difference of tumor volume in CA4P + nab-paclitaxel group was found compared with that of the PBS, CA4P and nab-paclitaxel group (*P* < 0.01) from day 14 on.

Tumor volumes on day 0, 7, 14, 21 were shown in Figure 5. The tumor volumes at baseline were approximate in the PBS, nab-paclitaxel, CA4P and CA4P + nab-paclitaxel group, respectively (*P* > 0.05). There was no significant difference (*p* > 0.05) among the groups in tumor volume on day 7 after different treatments. However, on day 14, mean tumor volumes in PBS, nab-paclitaxel and CA4P group were 4.0, 3.0 and 2.3 times that of CA4P + nab-paclitaxel group, respectively. Sequential administration of CA4P and nab-paclitaxel showed significantly stronger tumor growth inhibition effect than that of administration of nab-paclitaxel alone on day 14 (1.88 ± 0.31 cm^3^ vs 5.71 ± 0.75 cm^3^, *p* < 0.01) and 21 (3.34 ± 0.56 cm^3^ vs 8.85 ± 0.94 cm^3^*p* < 0.01).

**Figure 6:**
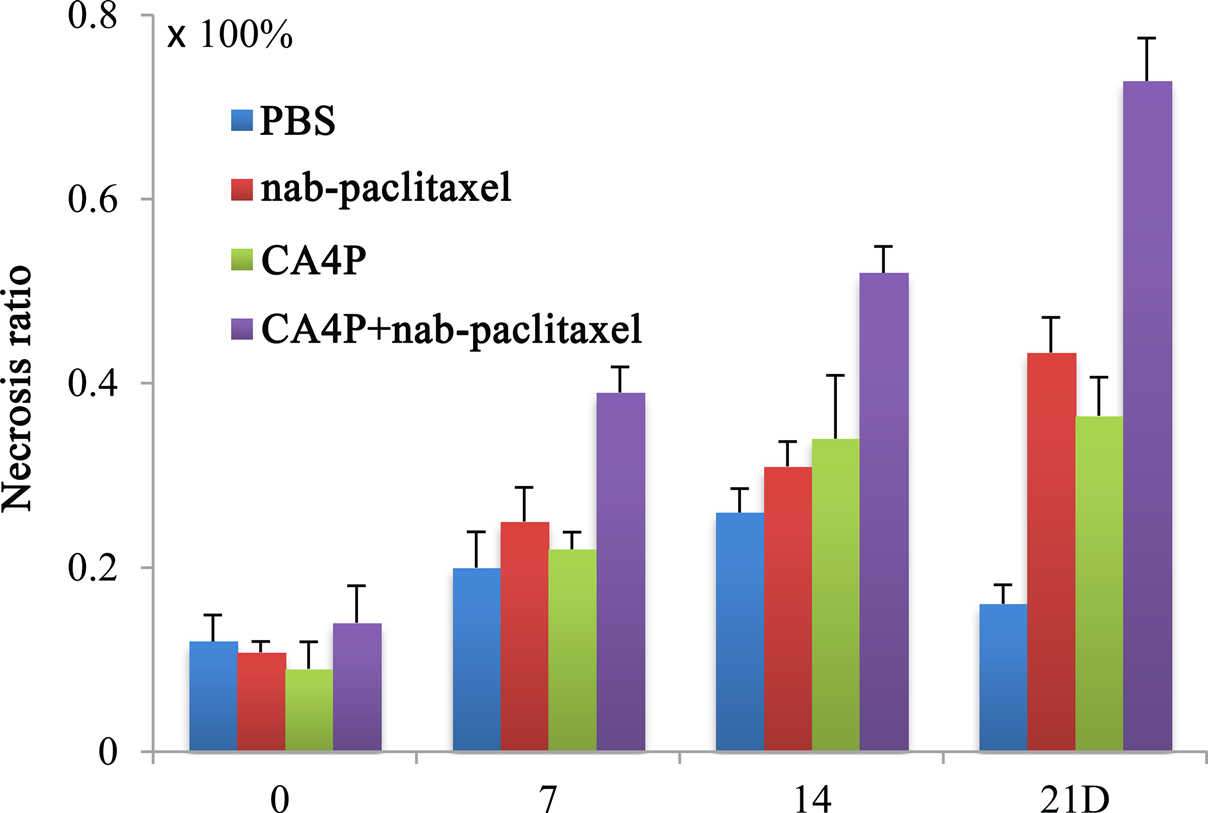
Tumor necrosis ratios on day 0, 7, 14 and 21 post-therapy A significantly higher necrosis ratio was observed in CA4P + nab-paclitaxel group compared with the PBS, CA4P and nab-paclitaxel group (*P* < 0.05) from day 7 on.

Tumor necrosis ratios on day 0, 7, 14, 21 were shown in Figure 6. Tumor necrosis measured from CE-T1W images at baseline was about 12% in each group because of the existence of spontaneous necrosis. After different treatments, a significantly higher necrosis ratio was observed in CA4P + nab-paclitaxel group compared with the PBS, CA4P and nab-paclitaxel group, respectively (*P* < 0.05) from day 7 till day 21. On day 21, the necrosis ratio was 16.24 ± 2.10%, 43.36 ± 3.79%, 36.52 ± 3.65% and 72.83 ± 4.64% for the PBS, nab-paclitaxel, CA4P and CA4P + nab-paclitaxel group, respectively, which further demonstrated the advantage of the coadministration of CA4P and nab-paclitaxel.

## DISCUSSION

PTX is one of the most effective drugs in the management of breast cancer [16, 17]. Although nanoparticle delivery of PTX improves the tumor uptake compared to free PTX [18, 19], the tumor response to the improved formulation of paclitaxel is typically only 30% to 35% [18–20]. The present work demonstrated that CA4P could improve the uptake and distribution of nab-paclitaxel in W256 breast carcinoma tumor, thereby enhancing the antitumor efficacy of nab-paclitaxel.

Our present study demonstrated that CA4P could significantly reduce IFP in W256 tumor in a nonlinear time-dependent manner within 60 min. This is consistent with previous study by Ley et al. [15], who found that IFP values declined continuously within 90 min following CA4P treatment in C3H mammary carcinoma tumors, however, no further IFP reductions were observed at later time points. Three hours after CA4P treatment, IFP levels of approximately 70% of controls was observed, which indicates that IFP does not recover within that period [14]. Significant reduction in IFP after CA4P treatment may be attributed to the decrease in microvascular pressure (MVP) which is the principal driving force of IFP [21]. CA4P induced intravascular clotting, vessel obstruction, or both could cause a rapid decrease in perfusion [15, 22]. Interruption of blood supply would decrease MVP inside large parts of the tumor vasculature, removing the source of fluid for filtration across the vascular membrane.

Although nab-paclitaxel could be preferentially delivered to tumors, the distribution of nab-paclitaxel in untreated tumor was heterogeneous and mainly located in the periphery of the tumor. This may correlate spatially with the peripheral IFP gradient as measured in subcutaneous OHS xenografts, where the high central IFP decreased rapidly in the periphery of the tumor [8]. In addition to high IFP, heterogeneous vascularization and perfusion together with slow interstitial diffusion may also contribute to the poor distribution [5].

CA4P could increase the tumor uptake and improve intratumor distribution of nab-paclitaxel. The present study indicated that not only was the uptake of nab-paclitaxel in tumor significantly higher, but also the distribution of nab-paclitaxel within tumor was more homogeneous in CA4P treated tumors compared with untreated tumors. This may be mainly attributed to the role of CA4P in reducing IFP, despite that an increase in tumor vascular permeability induced by CA4P [23, 24] would also contribute to the extravasation of macromolecule drugs from blood flow into tumor. As previous studies had shown that reducing IFP could improve the uptake and distribution of monoclonal antibody and liposomal doxorubicin in human osteosarcoma xenografts [8, 9].

There is an extensive clinical development program to evaluate the efficacy and safety of nab-paclitaxel in combination with several agents routinely used in breast cancer to further enhance antitumor efficacy [19]. Our study, for the first time, demonstrated the effectiveness of combination therapy with nab-paclitaxel and CA4P, a vascular disrupting agent in an experimental model of W256 breast cancer. The significant increase in the efficacy of tumor-bearing rats in the nab-paclitaxel/CA4P combined group compared with rats treated with single drugs shows great potential for implementing such strategy in clinics. The superior efficacy of nab-paclitaxel/CA4P combination therapy might be explained by the following mechanisms. First, vascular disrupting agent therapy can reduce IFP leading to increased nab-paclitaxel delivery and improved distribution within tumor, which means that a better antitumor effect can be obtained even excluding the extensive cell kill following the vascular shutdown achieved with CA4P. Second, CA4P combined with nab-paclitaxel may exert a synergistic effect by targeting two distinct cell populations. CA4P can eliminate cells in the central part of the tumor where the delivery of administered nab-paclitaxel is limited, whereas nab-paclitaxel is more likely to kill the actively proliferating cells in the peripheral viable rim that survive CA4P treatment.

There are still some limitations in the present study. Firstly, the effect of CA4P on MVP was not studied because the corresponding experimental instrument is currently not available for us. The transport of nanomedicines across vessel walls and in the tumor interstitium occurs mainly by convection [25, 26], which depends mainly on the pressure gradients between MVP and interstitial pressure [21]. Whether the increased tumor uptake and improved intratumor distribution of nab-paclitaxel is caused by a transcapillary pressure gradient induced by CA4P is still unknown for us. Secondly, we only investigated the effect of a dose of CA4P (30 mg/kg) on tumor IFP. The relationship between the effect of CA4P in reducing tumor IFP and its dosage has not been studied in detail. Related issues will be further explored in the follow-up study.

## MATERIALS AND METHODS

### Animals and tumor model

Adult male Sprague-Dawley rats (250–300 g) were provided by the Experimental Animal Center, Jiangsu Province Academy of Traditional Chinese Medicine (Nanjing, Jiangsu, China). The rat models of breast cancer were prepared by subcutaneously inoculating W256 tumor cells (5 × 10^6^) at a volume of 0.2 ml into the right flank region of rats. The longest (*L*) and shortest (*W*) tumor diameters (mm) were measured by digital caliper and used to calculate tumor volume (*V*) according to the formula: *V* = *(L × W^2^)/2*. Experiments were performed when tumors had reached a size of approximately 0.25 ~ 0.35 cm^3^. All the animal experiments were approved by the institutional animal care and use committee.

### Drug preparation and administration

CA4P (HuaMei technology Co., Ltd, Wuhan, China) was diluted in phosphate buffered saline (PBS) solution at a concentration of 18.75 mg/ml and injected i.v. at a dose of 30 mg/kg. Nab-paclitaxel (Celgene Corporation, Summit) was diluted in PBS solution at a concentration of 5 mg/ml and injected i.v. at a dose of 6 mg/kg.

The Iodogen coating method was used to radioiodinate nab-paclitaxel to form ^131^I-nab-paclitaxel. Nab-paclitaxel was dissolved in PBS to form 1 mg/ml solution. Radioiodination was initiated by adding nab-paclitaxel and Na^131^I solution (volume ratio, 5: 1) into Iodogen (1, 3, 4, 6-tetrachloro-3a, 6a-diphenylglycouril; Pierce Biotechnology, ZI Camp Jouven, France)-coated tube. Afterwards, the mixture was vortexed and incubated at 30°C for 3 ~ 5 min. The radiochemical yield was determined by TLC using 10% trichloroacetate (TCA) as mobile phase. Separation of ^131^I-nab-paclitaxel from unbound radioactive iodine was performed on Sephadex G-50, eluted with 0.05 M phosphate buffer (pH 7.5) and collected in 0.5 ml fractions. The agent was intravenously injected at a dose of 14.8 MBq/kg.

### *In vitro* stability

131I-nab-paclitaxel mixed with rat plasma (volume ratio, 1: 9) was incubated at 37°C. And then RCP of ^131^I-nab-paclitaxel was determined by TLC at 0.5, 1, 2, 6, 12 and 24 h, respectively.

### IFP measurements

The IFP was measured in subcutaneous tumors using the wick-in-needle technique [27]. In brief, a 23-gauge sensing needle was inserted into the center part of the tumor and connected to a pressure transducer (Changzhou Qianhong Bio-pharma Co., Ltd, Jiangsu, China) via polyethylene tubing filled with sterile heparinized PBS(70 units/ml). Pressures were monitored online using a PowerLab analogue-to-digital recording system (PowerLab/8S, AD Instruments, Hastings, UK). Fluid communication between the needle and the tumor tissue was tested by compressing and decompressing the tubing, and it was accepted when the IFP did not vary by more than 20%. After reaching a stable IFP value, a single dose of CA4P were administered into the tail vein. Control animals received an equal volume of PBS. Subsequent IFP measurements were made at 0, 5, 15, 30, 45, and 60 min post injection in the same tumors.

### Whole-body biodistribution and intratumoral distribution of ^131^I-nab-paclitaxel

Twelve W256 tumor-bearing rats were divided into two groups. CA4P+ ^131^I-nab-paclitaxel group received i.v. administration of ^131^I-nab-paclitaxel at 1 h post injection of CA4P, while ^131^I-nab-paclitaxel group received i.v. administration of ^131^I-nab-paclitaxel and an equal volume of PBS. At 24 h post injection of ^131^I-nab-paclitaxel, all rats were euthanized. Blood, lung, heart, spleen, pancreas, small intestine, kidney, skeleton, liver, muscle and tumor were sampled, weighted and then radioactivity was measured with an automatic γ-counter (WIZARD; 2470, Perkin Elmer, New York, USA). The results were expressed as percentage of the injected dose per gram of tissues (% ID/g).

The above tumor tissues were frozen in the cryotome (Shandon FSE, Thermo Fisher Scientific Co., USA) and cut into 30 μm sections along the long axis of the center. Autoradiograms of these slides were obtained by 12 h exposure using a high performance storage phosphor screen (Super resolution screen, Canberra-Packare, Ontario, Canada) and then the screen was read using a Phosphor Imager scanner (Cyclone^™^, Canberra-Packard) with Optiquant^™^ software. Subsequently, the same slices were stained with hematoxylin-eosin (H&E) and digitally photographed.

### Quantitation of intratumoral PTX by LC-MS/MS [28–30]

An LC-MS/MS assay was employed to determine the concentrations of PTX in the above tumor samples. After the counting, 20 mg of each tumor mass was collected. Ten times volume of methanol was added and homogenized. Then, 60 μl tissue homogenate was mixed with 20 μl of the internal standard (IS) docetaxel solution (50 μg/ml). Next, 3 ml of methyl tert-butyl ether (MTBE) was added to the mixture and vortexed for 5 min. After centrifugation at 3500 g for 5 min, the supernatant was collected and evaporated to dryness under a stream of nitrogen in a water bath at 35°C. The samples were diluted and injected onto the LC-MS/MS system (LC: waters ACQUITY and MS: waters synapt).

### Combinational therapy protocols

Twenty-four W256 tumor-bearing rats were randomized into four groups of six animals. The animals in each group received sequential intravenous injections at a 1 h interval at day 0, 6, 13 and 20. CA4P + nab-paclitaxel group received i.v. injection of CA4P and nab-paclitaxel; CA4P group received i.v. injection of CA4P and PBS; nab-paclitaxel group received i.v. injection of PBS and nab-paclitaxel; PBS group received twice i.v. injection of PBS. MRI was performed before administration and every 7 days after administration to monitor and quantify tumor volume and necrosis. At day 21 after MRI scanning, animals in all groups were sacrificed. Then all tumors were excised and cut into 5 μm frozen sections for postmortem histopathology verification.

### Magnetic resonance imaging

MRI was performed using a clinical 1.5T MR magnet (Echo speed; GE Co., NY) with a rat coil for rat studies. Under isoflurane gas anesthesia, T1-weighted (T1W) and T2-weighted (T2W) spin-echo multi-slice coronal images were acquired. Then contrast enhanced T1-weighted (CE-T1W) images were obtained immediately after i.v. administration of Gd-DTPA (Bayer Schering Pharma AG, Berlin, Germany) at 0.2 mmol/kg. The related parameters are described below: Field of view (FOV) = 100 mm × 100 mm; T1W: Sequence SE, TR/TE = 550 ms/24 ms; T2W: Sequence FSE, TR/TE = 2920 ms/88 ms; CE-T1W: Sequence SE, TR/TE = 550 ms/60 ms.

Quantifications of tumor area were done by manually delineating the outline of the tumor mass on each T2W MRI slice covering the whole tumor. Tumor volume was calculated using the equation: tumor volume = Σ [tumor area on each slice × (slice thickness)]. The area of central nonenhancing region was delineated from CE-T1W images to estimate necrosis. The ratios of necrosis were defined as the volume of necrosis over that of entire tumor, i.e. necrosis ratio = Σ (area of necrosis × slice thickness)/(area of whole tumor × slice thickness) × 100%.

### Statistical analysis

Numerical data were expressed as the mean ± standard deviation. Statistical analysis was carried out with SPSS for Windows software package (version 17.0; SPSS, Chicago, IL, USA). Two-tailed independent samples *t*-test was used to compare the biodistribution of ^131^I-nab-paclitaxel and the uptake of intratumoral PTX between ^131^I-nab-paclitaxel and CA4P + ^131^I-nab-paclitaxel group. For other comparisons, a one-way ANOVA was used to test differences among groups. *P* value less than 0.05 was considered to be significant difference.
